# Paraoxonase 1 gene polymorphisms are associated with an increased risk of breast cancer in a population of Chinese women

**DOI:** 10.18632/oncotarget.15911

**Published:** 2017-03-06

**Authors:** Junrong Wu, Min Fang, Xiaoping Zhou, Bo Zhu, Zhi Yang

**Affiliations:** ^1^ Department of Clinical Laboratory, The Affiliated Tumor Hospital of Guangxi Medical University, Nanning, 530021, Guangxi, China; ^2^ Department of Clinical Laboratory, The First Affiliated Hospital of Guangxi University of Chinese Medicine, Nanning, 530022, Guangxi, China; ^3^ Department of Nuclear Medicine, The Affiliated Tumor Hospital of Guangxi Medical University, Nanning, 530021, Guangxi, China

**Keywords:** breast cancer, paraoxonase 1, gene polymorphisms, menopausal

## Abstract

In this study, we explored associations between paraoxonase 1 (PON1) L55M and Q192R gene polymorphisms and the risk of breast cancer in 365 female breast cancer patients and 378 healthy controls from the Guangxi region of southern China. The LM heterozygous and MM homozygous genotypes, as well as M carrier status and M alleles, were associated with an increased risk of breast cancer. In addition, the M allele was associated with postmenopausal status and increased nodal involvement. In contrast, none of the Q192R genotypes or alleles were associated with a change in breast cancer risk, or with any of the clinicopathological parameters. These results indicate that PON1 L55M genetic polymorphisms may be associated with the risk of breast cancer and could potentially serve as useful genetic markers for tumor prognosis in some populations of Chinese women.

## INTRODUCTION

Breast cancer (BC) is the second most common cancer worldwide, as well as the most common cancer and cause of death among women [1, 2]. In China, BC is also one of the most common diseases and the most common cause of death in women [3]. Aging, family history of cancer, and certain behaviors [4], as well as dietary habits [5, 6], are risk factors for BC. Endogenous metabolites and exogenous carcinogens cause genetic damage [7], and mutations in oncogenes and tumor suppressor genes [8] may contribute to the etiology of BC. In addition, oxidative stress may play a critical role in cell proliferation and malignant conversion during the development of BC [9].

Because paraoxonase 1 (PON1), which is located on chromosome 7q21.3, not only decreases oxidative stress but is also implicated in the development of many cancers, investigators have assumed that PON1 polymorphisms might be associated with an increased risk of BC. PON1 expression and genotype distributions vary widely among different human populations [10]. Molecular studies have revealed two common functional single-nucleotide polymorphisms (SNPs), L55M and Q192R, in the coding region of the PON1 gene [10, 11]. Low PON1 activity has been consistently linked to an increased risk of disease, including gastric cancer [12], systemic lupus erythematosus [13], and angiocardiopathy [14]. In addition, a number of studies indicate that PON1 L55M and/or Q192R polymorphism(s) are associated with an increased risk of epithelial ovarian cancer [15], lung cancer [16], prostate cancer [15, 17, 18], and lymphoma [18].

The relationship between PON1 L55M and/or Q192R polymorphism(s) and the risk of BC has also been investigated in recent years. One meta-analysis [18] reported an association between PON1-192R and a decreased risk of BC, while two other meta-analyses [17, 19] did not find any relationship between PON1-192R and BC risk. A few possible explanations for these inconsistent results are as follows: (1) differences in PON1 expression and genotype distribution among the geographical and ethnic populations included in the studies may have impacted the results; (2) the sample sizes for both breast cancer cases and controls were small in some studies; and (3) patient characteristics differed among the studies. Additional investigations are therefore required to clarify the impact of these polymorphisms on the risk of BC. However, little data is currently available regarding the association between PON1/55 and 192 polymorphisms and BC risk in Chinese populations. For that reason, we conducted this study to accomplish the following goals: (1) examine the association between PON1 L55M and Q192R polymorphisms and the risk of BC in women in Guangxi province; (2) analyze associations between these polymorphisms and clinicopathological characteristics in BC patients to determine whether these variations are useful genetic markers of BC; (3) perform subgroup analysis based on menopausal status; (4) evaluate the association between the cooccurrence of both polymorphisms and the risk of BC; and (5) analyze linkage disequilibrium between the two SNPs.

## RESULTS

### Patient characteristics

Initially, 372 subjects were enrolled in the BC group and 381 were enrolled in the control group. Five patients who had previously been diagnosed with BC were excluded from the BC group. Two additional subjects were excluded from each group due to coronary heart disease (CHD), because the association between PON1/55 and 192 polymorphisms and the risk of CHD remains unclear. A final subject who was unable to give blood was excluded from the control group. Ultimately, PON1 polymorphisms were analyzed in the remaining 365 women with a clinical and histological diagnosis of BC (mean age 48 ± 9 years) and 378 age-matched healthy women (mean age 48 ± 8 years). Table 1 summarizes the clinicopathological features of the eligible subjects and potential risk factors for BC. Mean age, menopausal status, BMI classification, pregnancy status, and tobacco and alcohol consumption were similar in the two groups. A first-degree family history of BC was more common in the BC group (4.9%) than in the control group (0.8%) (*p* = 0.001).

**Table 1:** General characteristics of breast cancer patients and the normal controls

Characteristics	Cases (n=365)(%)	Controls(n=378)(%)	P-value
Age	48±9	48±8	0.758
Menopausal			
Premenopausal	195(53.4)	194(51.3)	0.607
Postmenopausal	170(46.6)	184(48.7)	
BMI(kg/m^2^)			
≤24.9	242(66.3)	261(69.0)	0.707
25.0-29.9	92(25.2)	86(22.8)	
≥30.0	31(8.5)	31(8.2)	
Lymph node status			
N0	162(44.4)		
N1	203(55.6)		
AJCC stage			
I	18(4.9)		
II	113(31.0)		
III	178(48.8)		
IV	56(15.3)		
First-degree family history of breast cancer			
No	347(95.1)	375(99.2)	0.001
Yes	18(4.9)	3(0.8)	
Ever been pregnant			
No	350(95.9)	366(96.8)	0.559
Yes	15(4.1)	12(3.2)	
Smoking status			
No	337(92.3)	358(94.7)	0.232
Yes	28(7.7)	20(5.3)	
Alcohol drinker			
No	332(91.0)	350(92.6)	0.426
Yes	33(9.0)	28(7.4)	
Case by stage			
Local	133(36.4)		
Advanced	232(63.6)		
Estrogen Receptor(ER) status			
ER+	105(28.8)		
ER-	260(71.2)		
Progesterone Receptor(PgR) status			
PgR+	117(32.1)		
PgR-	248(67.9)		

BMI, body mass index; AJCC, American joint committee on cancer.

### L55M polymorphism and BC risk

The genotype frequencies of PON1 L55M conformed to Hardy–Weinberg equilibrium (HWE) in both the BC group (*p* = 0.095) and the control group (*p* = 0.139). The L55M polymorphism genotypes and allele frequencies differed between the BC patients and control subjects both in the overall analysis and in the subgroup analysis based on menopausal status. The distribution of the LL genotype differed from that of the LM and MM genotypes in the overall analysis (χ^2^ test with Bonferroni correction for multiple comparisons, *p* = 0.017). The frequency of the LL genotype also differed from that of the LM genotype in the subgroup analysis (χ^2^ test with Bonferroni correction). In addition, the frequency of LL differed from that of the M carrier in both the overall and subgroup analyses. We then used the LL wild-type genotype and the L wild-type allele as references to analyze the risk of developing BC. The heterozygous LM mutation (OR_adj_ = 2.93, 95% CI 1.86–4.61, *p* < 0.001), the homozygous mutant genotype MM (OR_adj_ = 5.57, 95% CI 1.19–26.04, *p* = 0.029), and the M carrier (OR_adj_ = 3.09, 95% CI 1.99–4.79, *p* < 0.001) or M allele genotype (OR_adj_ = 3.00, 95% CI 1.99–4.51, *p* < 0.001) were associated with an increased risk of BC.

In the subgroup analysis based on menopausal status, the observed L55M genotype frequencies were in HWE (BC group, P = 0.641; control group, *p* = 0.657) in premenopausal subjects. Because the MM genotype was not observed in controls, the association between MM and BC risk could not be examined. Subjects with the LM genotype (OR_adj_ = 2.63, 95% CI 1.29–5.36, *p* = 0.007) and the M carrier (OR_adj_ = 2.79, 95% CI 1.38–5.64, *p* = 0.004) or M allele genotype (OR_adj_ = 2.79, 95% CI 1.41–5.49, *p* = 0.003) had an increased risk of BC. L55M genotype frequencies in the postmenopausal group also conformed to HWE (BC group, *p* = 0.183; control group, *p* = 0.078). Women with the LM genotype (OR_adj_ = 3.42, 95% CI 1.86–6.23, *p* < 0.001) and the M carrier (OR_adj_ = 3.57, 95% CI 2.00–6.37, *p* < 0.001) or M allele genotype (OR_adj_ = 3.28, 95% CI 1.95–5.53, *p* < 0.001) had an increased risk of BC. Individuals with the MM homozygous genotype tended to have a lower risk for BC (OR_adj_ = 4.96, 95% CI 1.00–6.37, *p* = 0.050), although this difference was marginally significant. The L55M polymorphism genotype and allele frequencies for the BC and normal groups are listed in Table 2.

**Table 2:** Distribution of PON1 L55M allele and genotype frequencies in breast cancer group and the controls group

	Case,n(%)	Controls,n(%)	OR(95%CI)	*P*_R_	OR_adj_(95%CI)	*P*_OR_
**PON1 L55M**						
All						
LL	284(77.8)	346 (91.5)	1.00		1.00	
LM	72(19.7)	30 (7.9)	2.92(1.86-4.60)	0.000	2.93(1.86-4.61)	0.000
MM	9(2.5)	2 (0.5)	5.48(1.18-25.58)	0.030	5.57(1.19-26.04)	0.029
LM+MM	81(22.2)	32 (8.4)	3.08(1.99-4.78)	0.000	3.09(1.99-4.79)	0.000
Alleles	n=730	n=756				
L	640(87.7)	722(95.5)	1.00		1.00	
M	90(12.3)	34(4.5)	2.99(1.98-4.49)	0.000	3.00(1.99-4.51)	0.000
Menopausal status at diagnosis						
Premenopausal						
LL	163(44.7)	182 (48.1)	1.00		1.00	
LM	30(8.2)	12 (3.2)	2.79(1.38-5.63)	0.004	2.63(1.29-5.34)	0.007
MM	2(0.5)	0 (0.0)	-	-	-	-
LM+MM	32(8.7)	12 (3.2)	2.99(1.48-5.97)	0.002	2.79(1.38-5.64)	0.004
Alleles	n=390	n=388				
L	356(91.3)	376(96.9)	1.00		1.00	
M	34(8.7)	12(3.1)	2.99(1.53-5.87)	0.001	2.79(1.41-5.49)	0.003
Postmenopausal						
LL	121(33.4)	164 (43.4)	1.00		1.00	
LM	42(11.5)	18 (4.8)	3.16(1.74-5.76)	0.000	3.42(1.86-6.28)	0.000
MM	7(1.9)	2 (0.5)	4.74(0.97-23.24)	0.055	4.96(1.00-24.56)	0.050
LM+MM	49(13.4)	20 (5.3)	3.32(1.88-5.88)	0.000	3.57(2.00-6.37)	0.000
Alleles	n=340	n=368				
L	284(83.5)	346(94.0)	1.00		1.00	
M	56(16.5)	22(6.0)	3.10(1.85-5.20)	0.000	3.28(1.95-5.53)	0.000

OR,odds ratio; OR_adj_, adjusted odds ratio; CI, confidence interval.

### PON1 Q192R polymorphism and BC risk

The distribution of Q192R genotypes was consistent with HWE in the BC group (*p* = 0.156) and the control group (*p* = 0.064). No significant differences were detected between the BC and control groups in the genotype frequencies of the QQ, QR, RR, and R alleles. The QR (OR_adj_ = 1.08, 95% CI 0.79–1.47, *p* = 0.645), RR (OR_adj_ = 1.05, 95% CI 0.68–1.63, *p* = 0.815), R carrier (OR_adj_ = 1.07, 95% CI 0.80-1.43, *p* = 0.648), and R allele (OR_adj_ = 1.04, 95% CI 0.84-1.29, *p* = 0.709) frequencies were not associated with BC risk. No relationship was found between BC risk and Q192R genotype or allele frequencies in either the premenopausal group or the postmenopausal group. The genotype and allele frequencies of the Q192R polymorphism for the BC and control groups are listed in Table 3.

**Table 3:** Distribution of PON1 Q192R allele and genotype frequencies in breast cancer group and the controls group

	Case,n(%)	Controls,n(%)	OR(95%CI)	*P*_OR_	OR_adj_(95%CI)	*P*_OR_
**PON1 Q192R**						
All						
QQ	155(42.5)	167 (44.1)	1.00		1.00	
QR	156(42.7)	156 (41.2)	1.08(0.79-1.47)	0.639	1.08(0.79-1.47)	0.645
RR	54(14.8)	55 (14.6)	1.06(0.69-1.63)	0.800	1.05(0.68-1.63)	0.815
QR+RR	210(57.5)	211 (55.8)	1.07(0.80-1.43)	0.637	1.07(0.80-1.43)	0.648
Alleles	n=730	n=756				
Q	466(63.8)	490(64.8)	1.00		1.00	
R	264(36.2)	266(35.2)	1.04(0.84-1.29)	0.694	1.04(0.84-1.29)	0.709
Menopausal status at diagnosis						
Premenopausal						
QQ	86(23.6)	86 (22.7)	1.00		1.00	
QR	81(22.2)	81 (21.4)	1.00(0.65-1.54)	1.000	0.97(0.63-1.50)	0.900
RR	28(7.7)	27 (7.1)	1.04(0.57-1.90)	0.907	0.92(0.50-1.72)	0.804
QR+RR	109(29.9)	108 (28.5)	1.01(0.68-1.51)	0.964	0.96(0.64-1.44)	0.847
Alleles	n=390	n=388				
Q	253(64.9)	253(65.2)	1.00		1.00	
R	137(35.1)	135(34.8)	1.02(0.76-1.36)	0.922	0.96(0.71-1.30)	0.800
Postmenopausal						
QQ	69(18.9)	81 (21.4)	1.00		1.00	
QR	75(20.5)	75 (19.8)	1.17(0.75-1.85)	0.488	1.23(0.77-1.94)	0.386
RR	26(7.1)	28(7.4)	1.09(0.59-2.03)	0.786	1.15(0.61-2.17)	0.656
QR+RR	101(27.6)	103(27.2)	1.15(0.76-1.76)	0.514	1.21(0.79-1.85)	0.392
Alleles	n=340	n=368				
Q	213(62.6)	237(64.4)	1.00		1.00	
R	127(37.4)	131(35.6)	1.08(0.79-1.46)	0.628	1.12(0.82-1.52)	0.488

OR,odds ratio; OR_adj_, adjusted odds ratio; CI, confidence interval.

### Relationships between L55M and Q192R polymorphism genotype frequencies and clinicopathological parameters in BC patients

Associations between L55M or Q192R polymorphisms, well-established prognostic parameters of BC, and the following clinicopathological characteristics were examined: age, menopausal status, BMI classification, lymph node status, American Joint Committee on Cancer (AJCC) stage, first-degree family history of breast cancer, pregnancy status, tobacco and alcohol consumption, case stage, and estrogen receptor (ER) and progesterone receptor (PgR) status. M carriers (LM+MM) in the BC group were more likely to be postmenopausal (*p* = 0.015) and have positive lymph node status (*p* < 0.001). M carrier genotypes were also associated with positive lymph node status in both the premenopausal and postmenopausal groups. In contrast, there were no significant associations between R allele genotype (QR+RR) carrier status and any of the clinicopathological parameters (Tables 4 and 5).

**Table 4:** Association between PON1 L55M polymorphism genotype frequencies and clinic-pathological parameters of the breast cancer patients

		Age	Menopausal		BMI(kg/m^2^)			Lymph node status			AJCC stage		
			Premenopausal	Postmenopausal	≤24.9	25.0-29.9	≥30.0	N0	N1	I	II	III	IV
ALL	LL	48 ± 10	163(57.4)	121(42.6)	183 (64.4)	73 (25.7)	28 (9.9)	140 (49.3)	144 (50.7)	14 (4.9)	89 (31.3)	134 (47.2)	47 (16.5)
	M	49 ± 9	32(39.5)	49(60.5)	59 (72.8)	19 (23.5)	3 (3.7)	22 (27.2)	59 (72.8)	4 (4.9)	24 (29.6)	44 (54.3)	9 (11.1)
*P*		0.231	0.015		0.180			0.000			0.807		
Premenopausal	LL	41 ± 5			96 (58.9)	52 (31.9)	15 (9.2)	106 (65.0)	57 (35.0)	12 (7.4)	54 (33.1)	68 (41.7)	29 (17.8)
	M	39 ± 6			20 (62.5)	10 (31.3)	2 (6.3)	13 (40.6)	19 (59.4)	3 (9.4)	8 (25.0)	16 (50.0)	5 (15.60)
*P*		0.313			0.485			0.001			0.174		
Postmenopausal	LL	57 ± 5			87 (71.9)	21 (17.4)	13 (10.7)	34 (28.1)	87 (71.9)	2 (1.7)	35 (28.9)	66 (54.5)	18 (14.9)
	M	55 ± 4			39 (79.6)	9 (18.4)	1 (2.0)	9 (18.4)	40 (81.6)	1 (2.0)	16 (32.7)	28 (57.1)	4 (8.2)
*P*		0.284			0.420			0.028			0.731		

		First-degree family history of breast cancer		Ever been pregnant		Smoking status		Alcohol drinker		Case by stage		Estrogen Receptor(ER) status		Progesterone Receptor(PgR) status	
		No	Yes	No	Yes	No	Yes	No	Yes	Local	Advanced	ER+	ER-	PgR+	PgR-
ALL	LL	270 (95.1)	14 (4.9)	274 (96.5)	10 (3.5)	263 (92.6)	21 (7.4)	259 (91.2)	25 (8.8)	109 (38.4)	175 (61.6)	79 (27.8)	205 (72.2)	93 (32.7)	191 (67.3)
	M	77 (95.1)	4 (4.9)	76 (93.8)	5 (6.2)	74 (91.4)	7 (8.6)	73 (90.1)	8 (9.9)	24 (29.6)	57 (70.4)	26 (32.1)	55 (67.9)	24 (29.6)	57 (70.4)
*P*		0.857		0.313		0.427		0.692		0.216		0.155		0.327	
Premenopausal	LL	154 (94.5)	9 (5.5)	154 (94.5)	9 (5.5)	143 (87.7)	20 (12.3)	141 (86.5)	22 (13.5)	90 (55.2)	73 (44.8)	48 (29.4)	115 (70.6)	58 (35.6)	105 (64.4)
	M	30 (93.8)	2 (6.3)	28 (87.5)	4 (12.5)	26 (81.3)	6 (18.8)	27 (84.4)	5 (15.6)	14 (43.8)	18 (56.3)	12 (37.5)	20 (62.5)	12 (37.5)	20 (62.5)
*P*		0.577		0.215		0.616		0.951		0.989		0.103		0.275	
Postmenopausal	LL	116 (95.9)	5 (4.1)	120 (99.2)	1 (0.8)	120 (99.2)	1 (0.8)	118 (97.5)	3 (2.5)	19 (15.7)	102 (84.3)	31 (25.6)	90 (74.4)	35 (28.9)	86 (71.1)
	M	47 (95.9)	2 (4.1)	48 (98.0)	1 (2.0)	48 (98.0)	1 (2.0)	46 (93.9)	3 (6.1)	10 (20.4)	39 (79.6)	14 (28.6)	35 (71.4)	12 (24.5)	37 (75.5)
*P*		0.455		0.291		0.559		0.129		0.858		0.347		0.340	

BMI, body mass index; AJCC, American joint committee on cancer.

**Table 5:** Association between PON1 Q192R polymorphism genotype frequencies and clinic-pathological parameters of the breast cancer patients

		Age	Menopausal		BMI(kg/m^2^)			Lymph node status			AJCC stage		
			Premenopausal	Postmenopausal	≤24.9	25.0-29.9	≥30.0	N0	N1	I	II	III	IV
ALL	LL	50 ± 9	71(45.8)	84(54.2)	101(65.2)	43(27.7)	11(7.1)	63 (40.6)	92 (59.4)	4(2.6)	44 (28.4)	85 (54.8)	22 (14.2)
	M	46 ± 9	124(59.0)	86(41.0)	141(67.1)	49(23.3)	20(9.5)	99 (47.1)	111 (52.9)	14(6.7)	69 (32.9)	93 (44.3)	34 (16.2)
*P*		0.413	0.103		0.395			0.859			0.326		
Premenopausal	LL	42 ± 6			41(58.6)	23(32.9)	6(8.6)	39 (55.7)	31 (44.3)	3(4.3)	19 (27.1)	36 (51.4)	12 (17.1)
	M	40 ± 5			75(61.0)	38(30.9)	11(8.9)	78 (63.4)	45 (36.6)	12(9.8)	42 (34.1)	48 (39.0)	22 (17.9)
*P*		0.137			0.642			0.182			0.116		
Postmenopausal	LL	57 ± 5			60(71.4)	19(22.6)	5(6.0)	23 (27.4)	61 (72.6)	1(1.2)	24 (28.6)	49 (58.3)	10 (11.9)
	M	56 ± 5			66(76.7)	11(12.8)	9(10.5)	20 (23.3)	66 (76.7)	2(2.3)	27 (31.4)	45 (52.3)	12 (14.0)
*P*		0.527			0.369			0.212			0.792		

		First-degree family history of breast cancer		Ever been pregnant		Smoking status		Alcohol drinker		Case by stage		Estrogen Receptor(ER) status		Progesterone Receptor(PgR) status	
		No	Yes	No	Yes	No	Yes	No	Yes	Local	Advanced	ER+	ER-	PgR+	PgR-
ALL	LL	147(94.8)	8(5.2)	147 (94.8)	8(5.2)	146 (94.2)	9 (5.8)	142 (91.6)	13 (8.4)	56 (36.1)	99(63.9)	48(31.0)	107 (69.0)	56 (36.1)	99(63.9)
	M	200(95.2)	10(4.8)	203 (96.7)	7(3.3)	191 (91.0)	19 (9.0)	191 (91.0)	19 (9.0)	77 (36.7)	133(63.3)	57(27.1)	153 (72.9)	62 (29.5)	148(70.5)
*P*		0.828		0.297		0.664		0.296		0.421		0.882		0.364	
Premenopausal	LL	66(94.3)	4(5.7)	63 (90.0)	7(10.0)	62 (88.6)	8 (11.4)	59 (84.3)	11 (15.7)	39 (55.7)	31(44.3)	23(32.9)	47 (67.1)	30 (42.9)	40(57.1)
	M	117(95.1)	7(5.7)	118 (95.9)	6(4.9)	106 (86.2)	18 (14.6)	108 (87.8)	16 (13.0)	64 (52.0)	60(48.8)	36(29.3)	88 (71.5)	39 (31.7)	85(69.1)
*P*		0.464		0.154		0.668		0.187		0.763		0.989		0.220	
Postmenopausal	LL	80(95.2)	4(4.8)	83 (98.8)	1(1.2)	83 (98.8)	1 (1.2)	81 (96.4)	3 (3.6)	16 (19.0)	68(81.0)	24(28.6)	60 (71.4)	24 (28.6)	60(71.4)
	M	83(96.5)	3(3.5)	85 (98.8)	1(1.2)	85 (98.8)	1 (1.2)	83 (96.5)	3 (3.5)	13 (15.1)	73(84.9)	21(24.4)	65 (75.6)	23 (26.7)	63(73.3)
*P*		0.436		0.946		0.898		0.427		0.680		0.765		0.869	

BMI, body mass index; AJCC, American joint committee on cancer.

### Linkage disequilibrium analysis and risk evaluation for both polymorphisms together

Results obtained from SHEsis software suggested that the two SNPs (L55M and Q192R) are not in strong linkage disequilibrium. The risk of BC was not affected when cooccurrence of both polymorphisms was examined using logistic regression analysis.

## DISCUSSION

In this study, we found that the frequencies of the LM, MM, and M alleles were higher in BC patients than in controls, which is consistent with previous studies [20, 21]. Additionally, our use of the Bonferroni correction for multiple tests, which resulted in more stringent criteria for statistically significant *p*-values, might have limited our ability to detect some interesting differences; logistic regression analysis may therefore be more appropriate for exploring the association between PON1 L55M polymorphisms and BC risk. Compared to wild-type homozygotes, women with LM alleles had a 2.93-fold increased risk of BC, and those who were MM homozygous had a 5.72-fold increased risk of BC. In addition, patients who were M carriers or with M alleles had a 3.09-fold and a 3.00-fold increased risk of BC, respectively. Furthermore, the risk of BC was higher in MM homozygous individuals than in those who were carriers of at least one allele (LM, LM+MM, and M allele). These results are consistent with those obtained by Antognelli *et al*. in a study of Italian BC patients [22]. However, in that study, the M carrier genotype was much more common in both the BC and control groups than we observed here (BC group: 80.4% vs. 22.2%; control group: 65.4% vs. 8.4%); this difference was even more striking among individuals who were MM homozygous (BC group: 59.4% vs. 2.5%; control group: 42.4% vs. 0.5%) [22]. These differences demonstrate the large degree to which the PON1 genotype distribution can vary among patients from different regions and of different ethnicities. Data from the haplotype map (HapMap) database indicates that ethnic populations native to western China are more prone to M mutations than populations native to eastern China. In this study, only 8.4% of control group patients were M carriers, which is consistent with data for the Han Chinese in Beijing (HCB) population in the HapMap database. Studies in American and Egyptian patients also indicate that women with the M allele of the L55M polymorphism might be at greater risk of BC, although they did not find that LM heterozygotes were at increased risk [20, 23]. Two previous meta-analyses [15, 19] also found that M allele frequency is positively correlated with an increased risk of BC.

In the subgroup analysis based on menopausal status, there were no healthy individuals with the MM genotype in the premenopausal group; a larger control group sample size is therefore needed to examine the relationship between this genotype, menopausal status, and BC risk. Antognelli *et al*. [22] found that the 35.7% of premenopausal women in their study who had the MM genotype were at a 3.83-fold higher risk of BC compared to control individuals; however, they did not find any increase in risk for patients with the LM genotype. Those authors also found that the MM frequency among their postmenopausal patients was higher than that observed here (47.5% vs. 1.9%), and the LL and MM genotypes as well as M carrier status were obviously associated with an increased risk of BC in their postmenopausal patients [22]. In our study, the association between MM homozygous genotype and BC risk was relatively weak, but it seems likely that studies with a larger sample size would confirm the existence of this relationship in Chinese women.

We did not find and significant associations between PON1 Q192R allele and genotype frequencies and BC risk in either the BC group or the control group, and Naidu *et al*. [21] and Hussein *et al*. [20] reported similar results. In contrast, Gallicchio *et al*. [24] found that PON1 Q192R polymorphisms are associated with a decreased risk of developing invasive BC, and Antognelli *et al*. [22] reported that the R allele is associated with a decreased risk of developing BC. In subgroup analysis, the latter study found that this relationship between the Q192R R allele and reduced BC risk was observed only in the postmenopausal group [22], implying that the PON1 R allele does not decrease the risk of BC in premenopausal women. In the present study, we did not detect a relationship between Q192R polymorphisms and risk of BC in either the premenopausal or postmenopausal groups. The differences between our results and those of Gallicchio *et al*. [24] and Antognelli *et al*. [22] again highlight the importance of ethnic background when examining the relationship between Q192R polymorphisms and BC risk. Naidu *et al*. [21], who also found no association between Q192R polymorphisms and BC risk, suggested that differences in BC patient and control group sample sizes among studies might contribute to discrepant results. However, we believe that ethnic differences are the main reason for this discrepancy. In recent years, meta-analyses [17–19] have explored the association between PON1 Q192R polymorphisms and the risk of BC. Fang *et al*. [17] and Wen *et al*. [19] both found that this polymorphism was not associated with the risk of BC, but Fang *et al*. [17] did find that the PON1-192R allele was associated with decreased risk of cancer in general in an Asian population. Although Zhang *et al*. [18] reported that the PON1-192R allele was associated with a decreased risk of BC, one of the studies included in that meta-analysis mistakenly reported genotype frequencies of 17%, 29%, and 6% for the QQ, QR, and RR alleles of PON1 Q192R, respectively, in the control group; the correct values are 6%, 29%, and 17%, respectively [25]. This error might be the primary source of these conflicting results. With this consideration in mind, previous studies and the current results suggest that the PON1-192R allele is not associated with an increase in susceptibility to BC in the Chinese population. Additional epidemiological studies in other regions and ethnic groups are needed to clarify this relationship in different patient populations.

In our examination of whether PON1 L55M or Q192R polymorphism genotype frequencies were associated with clinicopathological parameters in BC patients, we found that the PON1-55M allele was associated with postmenopausal status and lymph node metastases. In the subgroup analysis, the PON1-55M allele was also associated with lymph node status in both the premenopausal and postmenopausal groups. An association between the PON1-55M allele and positive lymph node status was also observed in two previous studies [20, 21]. In addition, Naidu *et al*. [21] noted that the PON1-55M allele was associated with the absence of ER; we observed a similar trend, but that difference did not reach statistical significance. In contrast, the association between the PON1-55M allele and postmenopausal status observed here has not been previously reported. It is well established that menopausal status is associated with susceptibility to BC; postmenopausal women are more likely to develop BC than premenopausal women. Xu *et al*. [26] demonstrated that menopausal status is a risk factor for BC in the Chinese population and the M variant may play an important role in facilitating tumor progression. In contrast, no correlation between the R allele genotype and clinicopathological parameters was found in this study or in previous studies.

In summary, this study is the first to explore the association between PON1 L55M and Q192R polymorphisms and the risk of BC in women in Guangxi, China. We found that PON1-55M variant was associated with an increased risk of BC in these patients and may play an important role in tumor progression. In contrast, the PON1-192R allele may not be a suitable marker for BC susceptibility and prognosis in this population. However, the small sample size in this study and the inability to analyze differences among women of different ethnicities in Guangxi province may limit the applicability of these results. Future studies with larger sample sizes and in women of different ethnicities are needed to confirm the utility of PON1 polymorphisms as genetic markers for the risk of developing BC and for tumor prognosis.

## MATERIALS AND METHODS

### Study population

Patients at the Affiliated Cancer Hospital of Guangxi Medical University and the First Affiliated Hospital of Guangxi University of Chinese Medicine were enrolled in this study between October 2013 and July 2015. Cancer patients with a previous history of BC or other diseases associated with PON1 L55M and/or Q192R polymorphisms were excluded. Modified criteria described by Bloom and Richardson [27] were used to determine grades for BC patients, and the AJCC staging system [28] was used to determine stages. Healthy control patients had no history of any malignancy or other diseases associated with PON1 L55M and/or Q192R polymorphisms. The “advanced” stage of BC was characterized by the presence of axillary lymph node-positive disease or metastatic BC at diagnosis, while the “local” stage was defined by a diagnosis of either *in situ* or invasive BC. The following demographic information was collected: age, menopausal status, BMI classification, lymph node status, AJCC stage, first-degree family history of BC, pregnancy status, alcohol and tobacco consumption, cases by stage, and ER and PgR status. All participants provided EDTA blood samples for genotyping. The study protocol followed the guidelines of the ethics committee of the Affiliated Cancer Hospital of Guangxi Medical University and the First Affiliated Hospital of Guangxi University of Chinese Medicine. All subjects provided informed consent regarding participation in the study.

### PON1 polymorphism screening

Genomic DNA was extracted from 2 mL of peripheral venous blood using a high salting-out method and phenol-chloroform. The PON1 L55M and Q192R SNPs were genotyped by polymerase chain reaction–restriction fragment length polymorphism (PCR-RFLP). The primers used for amplification of the Q192R polymorphism were as follows: forward, 5′-TATTGTTGCTGTGGGACCTGAG-3′; reverse, 5′-CACGCTAAACCCAAATACATCTC-3′ (99 bp). The primers used for amplification of the L55M polymorphism were as follows: forward, 5′-GAAGAGTGATGTATAGCCCCAG-3′; reverse, 5′-TTTAATCCAGAGCTAATGAAAGCC-3′ (170 bp). PCR amplification was performed in a final volume of 25 μL containing 1.0 μL of each primer, 12.5 μL of Green PCR Master Mix (Shanghai Sangon Biotech Co., Ltd., Shanghai, China), 9.5 μL of sterilized deionized water, and 2.0 μL of template DNA. The samples were amplified in a thermocycler (T100, Bio-Rad Laboratories, USA). After initial denaturation at 95°C for 5 min, the PCR reaction was exposed to 40 cycles of 95°C for 45 s, 59°C (Q192R segments) or 60°C (L55M segments) for 45 s, and 72°C for 45 s, followed by a final hold at 72°C for 10 min. The PCR products were separated on 2.5% agarose gels and subsequently stained with ethidium bromide for visualization. RFLP analysis was performed to detect SNPs after PCR amplification. The digested fragments were separated on 3% agarose and visualized using the Bio-Rad GelDoc 2000 XR+ System (Bio-Rad Laboratories). A negative control was included in each run to ensure the accuracy of genotype assessment.

### Statistical analysis

Hardy-Weinberg equilibrium (HWE) was assessed using a Pearson two-sided chi-square (χ^2^) test to explore the association between the observed and the expected numbers of each genotype in a population; *p* > 0.05 indicated conformance to HWE. When comparing differences in clinicopathological characteristics between BC patients and normal controls, χ^2^ tests were used for categorical variables and two-sample *t*-tests were used for continuous variables. χ^2^ tests with Bonferroni correction for multiple tests for statistically significant differences, as applicable, were used to evaluate differences in genotype and allele frequencies between the BC and control groups. The risk of BC was calculated using univariate logistic regression analysis, and crude odds ratio (OR) and 95% CIs were calculated for genotypes or alleles alone. Adjusted odds ratio (OR_adj_) and 95% CIs with an adjustment for age were calculated using the multivariate logistic regression method. Associations between genotype frequencies and clinicopathological parameters were determined using the multivariate logistic regression method. For all tests, *p* < 0.05 was considered statistically significant. Linkage disequilibrium between the two SNPs was analyzed using SHEsis software (http://analysis.bio-x.cn/myAnalysis.php). Logistic regression analysis was conducted to evaluate the risk for the two polymorphisms in combination. All statistical analyses were performed using the Statistical Package for Social Sciences, version 19.0 (SPSS Inc., Chicago, IL).
